# Antibiotic prescribing for upper respiratory infections among children in rural China: a cross-sectional study of outpatient prescriptions

**DOI:** 10.1080/16549716.2017.1287334

**Published:** 2017-05-02

**Authors:** Zhitong Zhang, Yanhong Hu, Guanyang Zou, Mei Lin, Jun Zeng, Simin Deng, Rony Zachariah, John Walley, Joseph D. Tucker, Xiaolin Wei

**Affiliations:** ^a^ China Global Health Research and Development, Shenzhen, China; ^b^ School of Public Health and Primary Care, Chinese University of Hong Kong, Hong Kong, China; ^c^ School of Public Health, Sun Yat-sen University, Guangzhou, China; ^d^ Guangxi Zhuang Autonomous Region Center for Disease Prevention and Control, Nanning, China; ^e^ Medical Department, Operational Research Unit, Médecins sans Frontières, Brussels Operational Center, Luxembourg City, Luxembourg; ^f^ Nuffield Centre for International Health and Development, University of Leeds, Leeds, UK; ^g^ Institute for Global Health and Infectious Diseases, University of North Carolina at Chapel Hill, Chapel Hill, NC, USA; ^h^ Dalla Lana School of Public Health, University of Toronto, Toronto, ON, Canada

**Keywords:** Antibiotics prescribing behavior, township hospital, multiple use of antibiotics, broad-spectrum antibiotics, injectable antibiotics

## Abstract

**Background**: Overuse of antibiotics contributes to the development of antimicrobial resistance.

**Objective:** This study aims to assess the condition of antibiotic use at health facilities at county, township and village levels in rural Guangxi, China.

**Methods:** We conducted a cross-sectional study of outpatient antibiotic prescriptions in 2014 for children aged 2–14 years with upper respiratory infections (URI). Twenty health facilities were randomly selected, including four county hospitals, eight township hospitals and eight village clinics. Prescriptions were extracted from the electronic records in the county hospitals and paper copies in the township hospitals and village clinics.

**Results:** The antibiotic prescription rate was higher in township hospitals (593/877, 68%) compared to county hospitals (2736/8166, 34%) and village clinics (96/297, 32%) (*p* < 0.001). Among prescriptions containing antibiotics, county hospitals were found to have the highest use rate of broad-spectrum antibiotics (82 vs 57% [township], vs 54% [village], *p* < 0.001), injectable antibiotics (65 vs 43% [township], vs 33% [village], *p* < 0.001) and multiple antibiotics (47 vs 15% [township], vs 0% [village], *p* < 0.001). Logistic regression showed that the likelihood of prescribing an antibiotic was significantly associated with patients being 6–14 years old compared with being 2–5 years old (adjusted odds ratio [aOR] = 1.3, 95% CI 1.2–1.5), and receiving care at township hospitals compared with county hospitals (aOR = 5.0, 95% CI 4.1–6.0). Prescriptions with insurance copayment appeared to lower the risk of prescribing antibiotics compared with those without (aOR = 0.8, 95% CI 0.7–0.9).

**Conclusions:** Inappropriate use of antibiotics was high for outpatient childhood URI in the four counties of Guangxi, China, with the highest rate found in township hospitals. A significant high proportion of prescriptions containing antibiotics were broad-spectrum, by intravenous infusion or with multiple antibiotics, especially at county hospitals. Urgent attention is needed to address this challenge.

## Background

Overuse of antibiotics contributes to the development of antimicrobial resistance. In China, overuse of antibiotics for humans and animals has resulted in high levels of antibiotics being detected among human excreta [1]. On average, each Chinese resident takes 138 g of antibiotics per year, tenfold greater than the average in the U.S.A. [2]. Existing studies on antibiotics in China have focused on unnecessary prescribing of antibiotics in urban areas or eastern provinces [3,4] or antibiotic resistance in eastern provinces [5–7]. Up to now, there have been very few studies conducted in western rural provinces of China where the inappropriate use of antibiotics may be at a greater level due to the relatively weak health system [8]. China’s rural health facilities, including county hospitals, township hospitals and village clinics, treat over 70% of the Chinese population; thus understanding antibiotic prescriptions in rural health facilities is crucial [9].

Examining prescriptions for childhood upper respiratory infections (URI) is a commonly accepted strategy to evaluate rationality of antibiotic use, as most URI are viral where antibiotics do not shorten the duration of the URI or prevent complications [10]. However, antibiotic prescribing for childhood URI is still often seen in practice, ranging from 20–90% [11], the highest rates being reported in Africa and Asia [11,12]. Antibiotic prescribing among children with URI can be used as a ‘proxy’ to measure the prevalence of unnecessary antibiotic use at various levels of the health facilities [13].

The World Health Organization has developed rational medicine use indicators which also include a benchmark for evaluating antibiotic prescriptions [14]. We investigated the antibiotic prescribing profile for URI among children at health facilities at county, township and village levels in rural Guangxi Province, China by adopting these indicators. Specifically, we aim to examine the following: the number and percentage of prescriptions for children with URI that included an antibiotic; the number, type, and route of administration for antibiotics prescribed; and factors associated with prescribing antibiotics for childhood URI

## Methods

### Study design

It is a retrospective, cross-sectional study of outpatient URI prescriptions from rural health facilities.

### Setting

Guangxi is one of the poorest provinces in China, located in the southwest mountainous terrains bordering with Vietnam and Laos. It is an ethnically diverse region with a population of 52 million, and more than 70% of the population are living in rural areas [15]. China’s rural health system is organised around three tiers. The county hospital is the clinical centre in rural areas, while township hospitals and village clinics are the primary care facilities. Normally, a county contains 10–30 townships, covering a population of around 300,000 to 1,000,000. A township contains 10–30 villages. Each village has around 200–800 households. Since 2012, rural residents in Guangxi have been covered by the New Rural Cooperative Medical Scheme (NCMS). NCMS encourages people to seek healthcare at health facilities of lower levels. For medications and service within its coverage, NCMS pays 80–90% of outpatient costs in township hospitals and 50–70% of outpatient costs in county hospitals. However, there are various barriers. For example, there is a ceiling for outpatient claims of 15–23 USD (100–150 RMB) per capita per year. Any amount beyond the ceiling has to be paid by patients themselves [16]. Also, the antimicrobial resistance has been found to be severe in Guangxi with high resistance rates of streptococcus pneumoniae to erythromycin (96%), cefuroxime (70%) and cefotaxime (61%) [17].

A stratified random sampling method was employed. We first divided the 68 rural counties in Guangxi into eastern, western, southern and northern regions. In each region, we randomly selected one county using a computer-generated random number based on the list of all the counties in this region. Within each county, two township hospitals were randomly selected through the same computer system and one village clinic was randomly selected under each selected township again through the same method. In total, four county hospitals, eight township hospitals and eight village clinics were included.

### Study population

**Figure 1.**
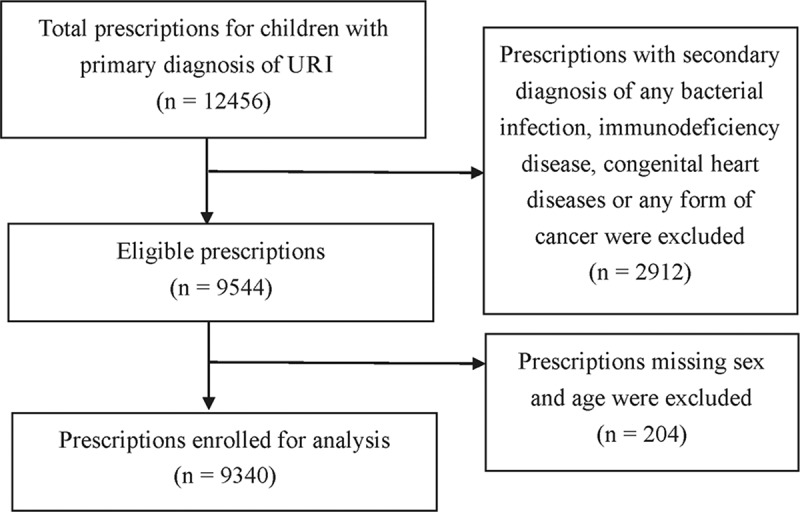
Flow chart of the enrolled prescriptions for children aged 2–14 with URI in rural Guangxi, China (2014).

We conducted a retrospective prescription review. Prescriptions in the 20 selected rural health facilities for children aged 2–14 years with a primary diagnosis of upper respiratory infection between January and December 2014 were included. Prescriptions were excluded if there was any diagnosis of a bacterial infection clearly requiring antibiotics (e.g. acute otitis media, pneumonia, tuberculosis). HIV/AIDS or other immunodeficiency diseases, congenital heart diseases and any form of cancer, requiring long-term antibiotic treatment or prophylaxis, were also excluded (see Figure 1). URI were defined according to the International Classification of Diseases (ICD), Revision 10, codes J00–J06 (J00 = Acute nasopharyngitis [common cold]; J01 = Acute sinusitis; J02 = Acute pharyngitis; J03 = Acute tonsillitis; J04 = Acute laryngitis and tracheitis; J05 = Acute obstructive laryngitis [croup] and epiglottitis; J06 = Acute upper respiratory infections of multiple and unspecified sites) [12].

### Data collection

Prescription data were either extracted from electronic data centres where available (in county hospitals), or photocopied from the written prescriptions using digital cameras (in all township hospitals and village clinics) by trained research assistants and medical students. All prescriptions were required to be recorded in the electronic system by doctors during clinic consultations in county hospitals. Diagnosis information was identified through an informatics search for the ICD code in county hospitals and manually screened in township hospitals and village clinics. Prescription data included patients’ basic information (age, sex, diagnosis and payment method), all the prescribed medicines, the prescribing date and the cost.

In China, a patient’s visit normally ends with a prescription because patients may feel they have not been taken seriously if they leave without a prescription [18]. Thus, each prescription represented a clinical consultation at all the health facilities. Prescriptions from repeated visits for a patient were also recorded as separate visits due to the lack of unique patient identifiers.

### Sample size

Based on preliminary field data, we estimated an antibiotic prescription rate of 45% for county hospitals, 50% for township hospitals and 70% for village clinics [5,6]. The minimum sample size of prescriptions was calculated as 380 for county hospitals, 384 for township hospitals and 323 for village clinics using the equation Z_1-__α/2_^_2_^p(1–p)/d^2^ with a precision/absolute error of 5% and type 1 error of 5% [19]. Where: Z_1-α/2_ = Standard normal variate. It is 1.96 at 5% type 1 error. p = Expected proportion in population based on previous studies. d = Absolute error or precision. We collected all the prescriptions in selected facilities to achieve this minimum requirement and avoid selection bias.

### Statistical analysis

The primary outcome indicator is the percentage of prescriptions that included antibiotics for children aged 2–14 years with a primary diagnosis of upper respiratory infection. Secondary outcome indicators were examined among prescriptions containing an antibiotic, including the percentage of those with an injection, or with combined antibiotics, or with broad-spectrum antibiotics. Quantitative variables were grouped by health facility level according to the research objective.

Amoxicillin-clavulanate, second- and third-generation cephalosporins, and azithromycin were considered broad-spectrum based on the classification in Steinman’s study [20]. In addition to these indicators, the percentages of prescriptions costing over 32 USD (200 RMB) and 8–32 USD (50–200 RMB) were calculated. A cost per prescription of over 32 USD was considered ‘over prescribed’ based on the Chinese regulation of outpatient prescription [21].

Data on copies of written prescriptions were double entered and validated using EpiData Entry software (version 3.1, EpiData Association, Odense, Denmark). Statistical analyses were conducted in Stata version 14 (StataCorp, College Station, TX). Missing values were not included for calculation of proportions. Differences between groups were assessed using the chi-square test for categorical data and *t*-test/ANOVA for continuous data. Factors associated with antibiotic use were examined using crude and adjusted odds ratios. Odds ratios were adjusted using binary logistic regression analysis and all *p*-values were based on the Wald test (*p* < 0.05). A step-wise backward regression process was used and included all variables with a *p*-value of less than 0.1 in the initial model.

## Results

### General characteristics of the collected prescriptions

Among the 9340 URI prescriptions enrolled for analysis, 8166 (87%), 877 (10%) and 297 (3%) were collected from county hospitals, township hospitals and village clinics respectively. More than half of the prescriptions were for boys and 76% were for children aged 2–5 years. Only 8% of the URI prescriptions were for URI of a single specified site and 92% were for acute URI of multiple or unspecified sites. Thirty-one per cent of the total eligible prescriptions had the insurance copayment, 43% were totally paid out-of-pocket, and 26% did not record information of payment method. The average number of medicines per prescription was 5 and the average cost per prescription was 14 USD (87 RMB). Prescription costs exceeding 32 USD were only seen at county hospitals. Seventy per cent of the prescriptions were given on working days and 30% were given on weekends (Table 1).

**Table 1.** General characteristics of prescriptions among children aged 2–14 with URI stratified by health facility level in Guangxi, rural China (2014).

	County hospital	Township hospital	Village clinic	Total
**Total URI prescriptions**	8166	877	297	9340
**Gender**				
Male	4769 (58)	491 (56)	156 (53)	5416 (58)
Female	3397 (42)	386 (44)	141 (48)	3924 (42)
Age group*				
2–5	6499 (80)	479 (55)	139 (47)	7117 (76)
6–14	1667 (20)	396 (45)	158 (53)	2223 (24)
**Diagnoses**^a^*				
J00	45 (< 1)	401 (46)	23 (8)	469 (5)
J01	3 (< 1)	0	0	3 (< 1)
J02	140 (2)	45 (5)	6 (2)	191 (2)
J03	17 (< 1)	59 (7)	16 (5)	92 (1)
J05	0	1 (< 1)	0	1 (< 1)
J06	7961 (98)	371 (42)	252 (85)	8584 (92)
Payment method*				
With insurance copayment	2354 (29)	285 (32)	232 (78)	2871 (31)
Fully out-of-pocket	3478 (43)	561 (64)	3 (1)	4042 (43)
Unknown	2334 (28)	31 (4)	62 (21)	2427 (26)
Average number of medicines*	6 (±5)	3 (±2)	2 (±1)	5 (±5)
Average prescription fee (USD)*	15 (±34)	4 (±3)	2 (±1)	14 (±5)
< 8	5570 (68)	761 (87)	295 (99)	6626 (71)
8–32	1560 (19)	116 (13)	2 (1)	1678 (18)
> 32	1036 (13)	0	0	1036 (11)
Date of prescription^b^**				
Working days	5683 (70)	521 (70)	192 (77)	6396 (70)
Weekends	2483 (30)	222 (30)	56 (23)	2761 (30)

Notes: Data are n (%) or mean (± standard deviation). ANOVA or chi-square test was used to find the statistical difference among health facilities of three levels: *p < 0.001; **p < 0.05.

J00 = Acute nasopharyngitis (common cold); J01 = Acute sinusitis; J02 = Acute pharyngitis; J03 = Acute tonsillitis; J05 = Acute obstructive laryngitis (croup) and epiglottitis; J06 = Acute upper respiratory infections of multiple and unspecified sites.

Missing values were excluded for proportion calculations: 134 (15.3%) and 49 (16.5%) missing values in township hospitals and village clinics for date of prescription.

### Antibiotics prescribing rate and characteristics of prescribed antibiotics

The percentage of antibiotic prescription was the highest in township hospitals (68%), almost two times higher than that in county hospitals (34%, *p* < 0.001) and village clinics (32%, *p* < 0.001) (Table 2). Among the prescriptions containing antibiotics in county hospitals, 47% contained multiple antibiotics, 82% had broad-spectrum antibiotics and 65% contained injectable antibiotics. Cephalosporin (I, II and III) was the most commonly prescribed antibiotic, followed by penicillin (first generation of penicillin: penicillin G, penicillin V, procaine penicillin and benzathine penicillin; second/third generation of penicillin). The most prescribed antibiotic was cephalosporin (74%) at county level (62 and 64% of these prescriptions included cephalosporin II and cephalosporin III), penicillin (51%) at township level and penicillin (33%)/aminoglycoside (33%) at village level. Only one prescription from a township hospital included a fluoroquinolone.

**Table 2.** Antibiotics prescribing rate and characteristics of prescribed antibiotics among children aged 2–14 with URI stratified by health facility level in rural Guangxi, China (2014).

	County hospital	Township hospital	Village clinic	Total
**Total URI prescriptions**	8166	877	297	9340
Total prescriptions with antibiotics*	2736 (34)	593 (68)	96 (32)	3425 (37)
No. of antibiotics prescribed*				
1	1438 (53)	506 (85)	96 (100)	2040 (59)
2	558 (20)	82 (14)	0	640 (19)
≥ 3	740 (27)	5 (1)	0	745 (22)
**Category of antibiotics**^a^				
Penicillin^b^***	1212 (44)	301 (51)	32 (33)	1545 (45)
Broad-spectrum penicillin	640 (53)	173 (58)	15 (47)	828 (54)
Narrow-spectrum penicillin*	695 (57)	131 (44)	17 (53)	843 (55)
Cephalosporin*	2019 (74)	208 (35)	28 (29)	2255 (66)
Cephalosporin I*	492 (24)	96 (46)	23 (82)	611 (27)
Cephalosporin II*	1257 (62)	57 (27)	2 (7)	1316 (58)
Cephalosporin III*	1300 (64)	75 (36)	3 (11)	1378 (61)
Macrolides**	238 (9)	37 (6)	4 (4)	279 (8)
Lincosamides*	109 (4)	59 (10)	0	168 (5)
Aminoglycosides*	10 (< 1)	40 (7)	32 (33)	82 (2)
Quinolones	0	1 (< 1)	0	1 (< 1)
Spectrum category of antibiotics*				
Narrow-spectrum only	502 (18)	255 (43)	44 (46)	801 (23)
Including broad-spectrum	2234 (82)	338 (57)	52 (54)	2624 (77)
Prescription type*				
Oral antibiotics only	956 (35)	336 (57)	64 (67)	1356 (40)
Including injectable	1780 (65)	257 (43)	32 (33)	2069 (60)
Average prescription fee (USD)*	38 (±50)	5 (±3)	2 (±1)	31 (±47)
< 8	653 (24)	484 (82)	96 (100)	1233 (36)
8–32	1067 (39)	109 (18)	0	1176 (34)
> 32	1016 (37)	0	0	1016 (30)

Notes: Data are n (%) or mean (± standard deviation). ANOVA or chi-square test was used to find the statistical difference among health facilities of three levels: *p < 0.001; **p < 0.05; ***p < 0.01.

More than one category of antibiotics may be prescribed in one prescription, thus the proportion may add up to more than 100%.

Penicillin included first generation of penicillin (penicillin G, penicillin V, procaine penicillin, and benzathine penicillin) and second/third generation of penicillin.

### Factors associated with antibiotic prescriptions among children with URI

Significant adjusted risk factors associated with antibiotic prescriptions included being aged 6–14 years old (aOR = 1.3, 95% CI 1.2–1.5) compared with younger children (2–5 years old), URI involving a single anatomical site (J00–J05) (aOR = 7.7, 95% CI 6.2–9.5) compared with multiple anatomical sites (J06), and receiving care at township hospitals (aOR = 5.0, 95% CI 4.1–6.0) compared with in county hospitals. Having insurance copayment appeared to lower the risk for prescribing antibiotics (aOR = 0.8, 95% CI 0.7–0.9) compared with those fully paid out-of-pocket (Table 3).

**Table 3.** Factors associated with antibiotic prescriptions among children aged 2–14 with URI in rural Guangxi, China (2014).

	Total URI prescriptions	Antibiotics prescribed, n (%)	Crude odds ratio	Adjusted odds ratio^a^	
				aOR (95% CI)	*p-*value
**Gender**					
Male	5416	1394 (38)	1.1 (1.0–1.2)	0.9 (0.8–1.0)	0.184
Female	3924	2031 (36)	Ref	Ref	
**Age**					
2–5 years	7117	2358 (33)	Ref	Ref	
6–14 years	2223	1067 (95)	1.9 (1.7–2.1)	1.3 (1.2–1.5)	< 0.001
**URI type^b^**					
URI of specified anatomical sites	756	581 (77)	6.7 (5.6–8.0)	7.7 (6.2–9.5)	< 0.001
URI of unspecified anatomical sites	8584	2844 (33)	Ref	Ref	
**Payment method**					
Unknown	2427	2073 (85)	20.0 (17.5–22.9)	38.4 (33.2–44.6)	< 0.001
With insurance copayment	2871	436 (15)	0.6 (0.5–0.7)	0.8 (0.7–0.9)	< 0.001
Fully out-of-pocket	4042	916 (23)	Ref	Ref	
**Health facility level**					
County hospital	8166	2736 (34)	Ref	Ref	
Township hospital	877	593 (68)	4.1 (3.6–4.8)	5.0 (4.1–6.0)	< 0.001
Village clinic	297	96 (32)	0.9 (0.7–1.2)	1.1 (0.8–1.6)	0.657

Notes: aOR was calculated using binary logistic regression.

URI of specified anatomical sites: acute nasopharyngitis (common cold), acute sinusitis, acute pharyngitis, acute tonsillitis, acute obstructive laryngitis (croup) and epiglottitis; URI of unspecified anatomical sites: acute upper respiratory infections of multiple and unspecified sites.

### Payment method and type of antibiotics prescribed

Of prescriptions with insurance copayment, 436 (15%) included antibiotics, which was significantly less than those that were paid out of pocket (n = 916, 23%, *p* < 0.001). More broad-spectrum antibiotics were found among antibiotic prescriptions with insurance copayment compared with those without (65 vs 55%, *p* < 0.01). The average cost for a prescription without insurance copayment was higher than for those with insurance copayment (4 ± 3 vs 5 ± 5 USD, *p* < 0.001). Prescription fees over 32 USD (> 200 RMB) were seen more frequently among prescriptions without insurance copayment (Table 4).

**Table 4.** Number, class and type of antibiotics prescribed among children aged 2–14 with URI stratified by payment method in rural Guangxi, China (2014).

	With insurance copayment	Fully out-of-pocket	Total
**Total URI prescriptions**	2871	4042	6913
Average prescription fee (USD)*	4 (±3)	5 (±5)	4 (±4)
< 8	2591 (90)	3508 (87)	6099 (88)
8–32	278 (10)	520 (13)	798 (12)
> 32	2 (< 0.1)	14 (< 1)	16 (< 1)
Prescriptions with antibiotics*	436 (15)	916 (23)	1352 (20)
**No. of antibiotics prescribed**			
1	396 (91)	831 (91)	1227 (91)
2	36 (8)	84 (9)	120 (9)
≥ 3	4 (1)	1 (< 0.1)	5 (< 0.1)
Spectrum category of antibiotics***			
Narrow-spectrum only	153 (35)	409 (45)	562 (42)
Including broad-spectrum	283 (65)	507 (55)	790 (58)
**Prescription type**			
Oral antibiotics only	268 (62)	525 (57)	793 (59)
Including injectable	168 (38)	391 (43)	559 (41)

Notes: Data are n (%) or mean (± standard deviation). *T*-test or chi-square test was used to find the statistical difference between payment method groups: *p < 0.001; ***p < 0.01.

## Discussion

This study shows a high antibiotics prescription rate for children aged 2–14 with URI in four counties of Guangxi, China. Many of these prescriptions included broad-spectrum antibiotics (54–82%). The prescribing rate of injectable antibiotics was also high (33–65%). The study’s strength is that we focused on a common childhood illness with access to prescriptions at all rural health facility levels. Importantly, this study addresses a national priority in China and provides evidence for policies and interventions to reduce antibiotic misuse.

We explored the reasons behind the main findings. First, we found a high rate of antibiotic use among pediatric URI prescriptions at township hospitals (68%), which is higher than in the studies from Song (44%) and Sun (58%) [5,22]. Song and Sun’s studies targeted wider populations rather than children. Part of the difference may also be explained by the heterogeneity of health practices in China. In 2012, China’s Ministry of Health (MOH) issued an antimicrobial stewardship policy of limiting to 20% outpatient prescriptions with antibiotics [23], but apparently, this did not achieve its target in our research setting. Primary care providers in township hospitals serve the majority of the rural population; however, many of these healthcare workers have less medical training, or were not sufficiently trained during their residency programmes [24]. Studies have also reported that clinicians from township hospitals attended less continuing education training on antimicrobial use compared with their peers in county hospitals [5]. This might partly explain why a higher antibiotic prescription rate was observed amongst the township hospitals.

Another possible reason for the high antibiotic prescribing rate is the common health beliefs among the Chinese public, including clinicians. As the studies by Sun and Reynolds found [5,25], even though the doctors were aware that antibiotics would not treat a viral infection, they still believed antibiotics might speed patient recovery. Provider-targeted and regular peer education on how to diagnose and treat URI correctly is important to improve prescribing practice [26]. Besides, the current tense relationship between clinicians and patients, including numerous cases of violence against clinicians in China, might also contribute to inappropriate prescribing behavior as clinicians are pushed by patients’ demands on antibiotics [27]. Qualitative research is needed to better understand how the social norms and standards of care that influence antibiotic prescribing behavior can be changed.

Second, a high proportion of antibiotics (60%) were injectable in this study. This was higher than the rate found in Li’s study (33–38%) [4]. Injectable antibiotic use via prescriptions exposes patients to unnecessary drug-related side effects, but also brings substantial financial burdens and medical risks. Moreover, additional injection equipment adds to the generation of medical waste. Such waste needs careful disposal and adds to health facility expenses and workload. Healthcare workers and patients may also be more exposed to blood-borne infections such as hepatitis B, C and HIV [28].

Third, we found a high rate of broad-spectrum molecules (54–82%) among antibiotics prescriptions, which is similar to the rate (72%) reported by a recent study [29]. The wide use of broad-spectrum antibiotics would be a challenge for reducing antimicrobial resistance, as shown in other studies [30,31]. This study indicated that nearly two-thirds of antibiotic prescriptions included cephalosporin II and cephalosporin III in county hospitals. This could potentially explain the high antimicrobial resistance rate to cefuroxime and cefotaxime in the research area [17]. Following the study, we have designed and implemented a standard package of education and a reminder system for township physicians to reduce antibiotic prescriptions [32].

Fourth, we found that prescriptions without insurance copayment were more likely to include antibiotics. This is consistent with a study from the U.S.A. and a pre-health reform study in China [33,34]. The majority of people in rural areas of China have been covered by the health insurance scheme, NCMS The ceiling for outpatient claims was around 23 USD per capita per year in 2015 in Guangxi, which was not enough to meet patients’ demands [35]. Patients have to pay any additional costs above the ceiling. We suppose two possible scenarios. First, patients who have already used up their insurance may postpone their care and only attend the clinic at later stages of illness when complications are more common [36]. Second, prescriptions are not adequately audited for antibiotic use or for costing purposes if they do not fall under the NCMS [4]. How to implement a rural insurance programme to avoid the incentive from medicine prescription remains a challenge for policy makers.

This study has several limitations. Firstly, we collected large numbers of prescriptions at the county level, but relatively small numbers at the township and village levels due to their availability. In the study interpretation, we stated results from each level to avoid the overwhelming weight of county-level prescriptions. Second, there is a possibility of misclassification of diagnosis under the ICD-10 coding [12]. However, the use of ICD-10 codes is well-established within health systems research, and this has been used for previous studies of URI [37]. Thirdly, URI may be complicated by other appropriate indications that need antibiotics. However, over 90% of acute URI are viral and only very few may be bacterial or lead to bacterial infections [13]. In addition, we excluded any complications or bacterial infections. Finally, we may include repeated visit data but the antibiotics rate estimation has been based on number of prescriptions rather than individual patients and so there would not be an issue of over-estimation. We cannot distinguish between initiative visits and follow-up visits.

Our study has implications for policies to reduce unnecessary antibiotics use. First, strengthening the supervision of antibiotic use in township hospitals is a priority. Second, limiting broad-spectrum and injectable antibiotic use is clearly needed. This will need operational policy on educational interventions for both clinicians and the general population [6]. Third, our data suggest that health systems factors and financial incentives may influence antibiotic over-prescribing. Fourth, further training for clinicians is required on clinical skills and their communication with patients. Evidence from systematic review shows that interventions that targeted both clinicians and patients for up to 3 months’ duration, particularly training on doctor–patient communication skills, showed better effects than individual interventions [38]. Interventions should improve prescribing behaviour, and rebuild patient–physician trust when not prescribing antibiotics.

## Conclusion

Antibiotics are prevalent in prescriptions for childhood URI at all three levels of healthcare facilities in four counties of Guangxi, China, with the highest rate reported in township hospitals. In all facilities, prescribing broad-spectrum antibiotics or use of intravenous infusion was common. Urgent attention is needed to address this challenge of unnecessary antibiotics use in western rural China.

## Paper context

Previous studies have reported high use of antibiotics in China’s urban areas. This study added the evidence of high unnecessary use of antibiotics in rural areas in Guangxi, China. Among the three-tiered rural health system, township hospitals were identified as having the highest rate of unnecessary antibiotics prescribing for outpatient childhood upper respiratory infections. This requires immediate policy attention for interventions as township hospitals provide the largest share of primary healthcare in rural China.
